# Parallel age‐related cognitive effects in autism: A cross‐sectional replication study

**DOI:** 10.1002/aur.2650

**Published:** 2021-12-04

**Authors:** Carolien Torenvliet, Annabeth P. Groenman, Tulsi A. Radhoe, Joost A. Agelink van Rentergem, Wikke J. Van der Putten, Hilde M. Geurts

**Affiliations:** ^1^ Dutch Autism & ADHD Research Center, Brain & Cognition, Department of Psychology University of Amsterdam Amsterdam The Netherlands; ^2^ Leo Kannerhuis, Autism Clinic (Youz/Parnassia Group) Amsterdam The Netherlands

**Keywords:** aging, autism, Bayesian analyses, cognition, gerontology, replication

## Abstract

Findings on age‐related cognitive effects in autism in adulthood are inconsistent across studies. As these studies substantially differ in their methodology, replication studies are needed. In this replication study frequentist (i.e., null‐hypothesis significance testing), and Bayesian statistics were used to investigate the hypothesis that in autistic adults compared to non‐autistic adults mostly parallel, but also protective age‐related cognitive effects can be observed. Participants were 88 autistic adults, and 88 non‐autistic matched comparisons (age range: 30–89 years, mean age: 55 years). Cognitive measures were administered on the following six domains: verbal memory, visual memory, working memory, Theory of Mind (ToM), verbal fluency, and processing speed, and self‐reported cognitive failures. Non‐autistic adults outperformed autistic adults on ToM, verbal fluency, and verbal memory, but only the first two were confirmed with Bayesian replication analyses. Also, more cognitive failures were reported by autistic adults. No interactions between group and age were observed, suggesting a parallel age‐related effect on all cognitive domains. In sum, previously observed difficulties in ToM and verbal fluency were replicated which seem to persist at older age. Previously reported parallel age‐related cognitive patterns were replicated, yet no evidence for protective age‐related patterns was found.

**Lay summary:**

We investigated whether our previous findings on cognitive aging in autism could be confirmed in a new study measuring the cognitive effects of age in autistic and non‐autistic adults. As expected, tasks that younger autistic adults had difficulties with (theory of mind, fluency) were also difficult for older autistic adults, and the effect of age itself was similar in autistic and non‐autistic adults. Unexpectedly, we observed no protective effects (less cognitive aging) in autism.

## INTRODUCTION

In the past decade, a growing body of literature investigated the cognitive performances of (older) adults with autism (Edelson et al., 2021; Wise, 2020). As children, adolescents, and young adults with autism have a different developmental trajectory on some cognitive domains (e.g., Hill, 2004), accelerated cognitive aging in autistic adults as compared to non‐autistic adults could be a concern (Bowler, 2007; Geurts & Vissers, 2012), especially given the higher prevalence of neurodegenerative disorders in autistic adults (Croen et al., 2015; Geurts, McQuaid, et al., 2020a), and the first hints that the autistic brain might indeed decline faster (Koolschijn et al., 2017). However, autistic adults^1^ could also be less susceptible to cognitive aging (i.e., a protective aging pattern) as they might have acquired certain cognitive strategies, which can be used at older age (Geurts & Vissers, 2012). On a neural level, this hypothesis is supported by preliminary evidence indicating that autistic adults have higher levels of cortical excitability (Oberman & Pascual‐Leone, 2014). Evidently, both patterns deserve further investigation, and either can be true for different cognitive domains.

Results on cognitive aging in autism have been inconsistent, showing evidence for more pronounced aging or less pronounced aging between studies (Lever & Geurts, 2016; Powell et al., 2017) or even within the same study (Abbott et al., 2018; Geurts & Vissers, 2012). When separating these results into different cognitive domains a clearer pattern can be observed: difficulties of younger autistic adults in processing speed and verbal fluency seem to stay largely the same at older age (Ambery et al., 2006; Davids et al., 2016; Geurts, Pol, et al., 2020b; Geurts & Vissers, 2012; Lever & Geurts, 2016). However, difficulties in memory become less evident with increasing age (visual memory; Lever & Geurts, 2016; Tse et al., 2019, but see Geurts & Vissers, 2012 and Ring et al., 2016) or were not that evident even in the first stages of adulthood (verbal memory; Braden et al., 2017; Lever & Geurts, 2016; Tse et al., 2019). Results on working memory are conflicting. Most studies observe slower response times, but no differences in accuracy between autistic and non‐autistic groups on working memory tasks in middle and late adulthood (Abbott et al., 2018; Braden et al., 2017; Geurts et al., 2020b). One study indicated a protective aging pattern for autism (Lever et al., 2015), yet other studies indicate poorer working memory performance in older autistic adults (Geurts & Vissers, 2012; Tse et al., 2019). Studies on Theory of Mind (ToM) in autistic adults indicate that the observed difficulties in ToM decrease with age, but only when analyzing age categorically (i.e., younger or older than 50 years; Lever & Geurts, 2016; Zivrali Yarar et al., 2020). Even though all three patterns (i.e., parallel, accelerated, and protective aging) warrant further investigation, most evidence seems in line with either a parallel or protective aging pattern of cognitive aging, in which initial differences between autistic and non‐autistic adults in cognitive functioning largely stay the same (i.e., parallel) or decrease when growing older (i.e., protective).

Notably, most of the abovementioned studies suffer from power issues. Overestimations of effect sizes might have occurred due to insufficient sample sizes. The work by Lever et al. (2015) and Lever and Geurts (2016) were the first high‐powered cross‐sectional study on cognitive aging in autism. These researchers showed that in autism older age was associated with poorer performance in all aspects of memory (visual, verbal, working), ToM and processing speed, but not verbal fluency. Compared to non‐autistic adults, Lever and colleagues observed mostly parallel (similar) aging, and even a protective (smaller) aging effect of autism for some functions (working memory, visual memory, and ToM). The current study aims to replicate these results (Lever et al., 2015; Lever & Geurts, 2016) with the use of a second, high‐powered cross‐sectional study covering the full adult age range, and using the same cognitive tasks as were used in the original study. In this way, the current study investigates which results on cognitive aging in autistic adults can be confirmed, without procedural differences between studies hindering study comparisons. Furthermore, Bayesian replication analyses (Ly et al., 2019; Verhagen & Wagenmakers, 2014) were performed, which uses the data from the original study as a prior for the current results—maximizing the use of prior knowledge. Based on the previous study by Lever and colleagues (Lever et al., 2015; Lever & Geurts, 2016), and studies with older adult samples (Davids et al., 2016; Geurts et al., 2020b; Geurts & Vissers, 2012; Tse et al., 2019) we expected: (1) poorer performance of the autism group than comparisons on ToM, fluency, and processing speed, better performance of the autism group than comparisons on visual memory, and equal performance on visual working memory (2) either a similar relation with age (verbal memory, fluency and processing speed) or a smaller negative relation with age (visual memory, working memory and ToM) for the autism group than comparisons.

## METHODS

The current study is a direct replication (for definition see: Schmidt, 2009) of a previous study (Lever et al., 2015; Lever & Geurts, 2016). Similar recruitment strategies, materials, and instruments for inclusion were used. All dependent measures were exactly the same as in the original study. A detailed description of the minor differences between the origin, and the (current) replication study is provided in the Supporting Information.

### Participants

Participants (*n* = 194; *n*_autism_ = 88 and *n*_comparison_ = 106) between 30 and 89 years were recruited via several clinical institutions across the Netherlands, (social) media advertisements of autism networks, and the social network of the researchers, research assistants, and students. The exclusion criteria for both groups were (1) a history of neurological disorders (e.g., epilepsy, stroke, multiple sclerosis), schizophrenia or having experienced more than one psychosis, (2) Wechsler Adult Intelligence Scale‐IV (WAIS‐IV; Wechsler, 2003) IQ <70 or Mini Mental State Examination (MMSE; Folstein et al., 1975) <18,^2^ (3) current alcohol or drugs dependency as indicated by the administration of the MINI International Neuropsychiatric Interview (MINI; Sheehan et al., 1997; Van Vliet et al., 2000). For the autism group, two additional exclusion criteria were (1) no registered diagnosis of autism according to the DSM (*American Psychiatric Association*, 2013) criteria, (2) scoring below the cut‐off on both the ADOS‐2 (social Affect ≤6 and/or total score ≤8), and the Autism Quotient (AQ, Baron‐Cohen et al., 2001) <26. For the comparison group four additional exclusion criteria were: (1) a history of autism or AD(H)D), (2) close family‐members with ASD or AD(H)D, 3) AQ > 32, (4) ADHD‐SR symptoms in childhood ≥7 and/or adulthood ≥6. Participants were matched on age, sex and IQ.

### Measures

A brief overview of the used measures can be found in Table 1. Details of each of the measures can be found in Lever et al. (2015), and Lever and Geurts (2016). All measures were considered to have sufficient psychometric properties, and have been previously used in aging as well as autism research.

**TABLE 1:** Overview of measures used in the current study

Domain	Measure	Outcome	Additional information (score range)
Verbal memory	RAVLT^a^	Verbal Recall I	Sum immediate recall trial 1–5 (0–75)
		Verbal Recall II	Delayed recall (0–15)
		Verbal Recognition	Total correct (0–30)
Visual memory	WMS‐III^b^	Visual Recall I	Immediate recall (0–104)
		Visual Recall II	Delayed recall (0–104)
		Visual Recognition	Total correct (0–48)
Visual working memory	N‐back^c^	Working memory	Accuracy difference score (−1.0–1.0)
Theory of Mind	Faux‐Pas^d^	Theory of Mind	Total score (0–38)
Fluency	DAT^e^	Letter Fluency	Nr. of correct words
	GIT^f^	Category Fluency	Nr. of correct words
Processing speed	CRT^g^	Processing speed	Mean reaction time
Subjective cognition	CFQ^h^	Subjective cognition	Total score (0–100)

^a^ Dutch version of the Rey Auditory Verbal Learning Task (RAVLT, Rey, 1964; Saan & Deelman, 1986).

^b^ Subtest visual reproduction of the Wechsler Memory Scale Third Edition (WMS‐III; Wechsler, 1997).

^c^ Proportion correct in a 2‐back compared to a 0‐back condition (in house development, Lever et al., 2015).

^d^ Short, Dutch version (9 stories) of the Faux‐Pas task (Baron‐Cohen et al., 1999; Spek et al., 2010).

^e^ Dutch version of the Controlled Oral Word Association Test (COWAT, Benton & Hamsher, 1989; Schmand et al., 2008).

^f^ Subtest Word Naming (animals and professions) of the Groninger Intelligence Test (GIT; Luteijn & Barelds, 2004).

^g^ 2‐choice response task, (in house development, Lever et al., 2015).

^h^ Cognitive Failures Questionnaire (CFQ, Broadbent et al., 1982).

### Procedure

The study is part of a larger study on aging in autism (Geurts et al., 2021), in which multiple independent cohorts are included at different time‐points. Our replication study cohort started in 2019, and data from the original study were collected in 2014. As aforementioned, the procedure was highly similar to the procedure described by Lever et al. (2015), Lever and Geurts (2016). The screening procedure and corresponding materials are described in the study protocol paper see Geurts et al. (2021). Cognitive tasks were administered during 2–2.5‐h testing sessions in counterbalanced order, so no test could have been systematically influenced by tiredness and/or practice. All participants received monetary compensation (€30,‐) for their participation, and (partial) reimbursement of their traveling costs. The study was approved by the ethical review board of the Department of Psychology of the University of Amsterdam (2018‐BC‐9285).

## ANALYSES

Our pre‐registered analysisplan (#40091) on AsPredicted.org was based on the neuropsychological test‐battery study of Lever and Geurts (2016), and two additions based on the working memory study of Lever et al. (2015). To measure group differences, MANOVA analyses for visual memory, verbal memory, and verbal fluency, were used and *t*‐tests^3^ for ToM, working memory, processing speed, and subjective cognition with group as a between‐subjects factor (autism vs. comparison). As non‐normality was observed in all test variables, non‐parametric results were reported (Wilcoxon rank‐sum tests). In addition to Lever and Geurts (2016) Bayes Factors on group differences to assess the strength of null results were reported (referred to as: “BFcurrent”). To assess the effect of age, linear multiple regression analyses were performed for each outcome variable, with (centered) Age, Group, and Age x Group as predictors. In addition to Lever and Geurts (2016) regression analyses with Age^2^ and Age^2^ x Group as predictors were reported to assess possible non‐linear effects. Lastly, group‐ and regression analyses were performed in a subgroup with only 50+ participants to assess these effects in older age separately.

In addition to our pre‐registered analyses, we used a Bayesian replication method proposed by Verhagen and Wagenmakers (2014), and Ly et al. (2019). This method is specifically designed to assess the replication strength when comparing means. We used the Bayesian replication method to assess the similarity between the current findings and those of Lever and Geurts (2016), and Lever et al. (2015). The replication method includes several merits of Bayesian analyses, namely that (1) Bayesian comparisons, opposed to *p*‐values, account for power differences between studies, which facilitates the comparison of results, (2) Bayesian analyses make use of previous results by explicitly including prior expectations (e.g., previous results) in the analyses, and (3) the Bayes Factor, a Bayesian estimate of effect size, assesses the strength of null results, and distinguishes between a lack of power, and a “true” null result. To ease interpretation, three important concepts to consider in Bayesian analyses are the prior distribution, the posterior distribution, and the Bayes Factor. The prior distribution defines the belief about the effect *before* (i.e., prior to) analysis of any data. When no prior belief is specified, a “flat” or “uninformative” prior can be used, not steering the data in any particular direction. The posterior distribution is dependend on the prior and the data, and indicates the belief about the effect after seeing the data. The peak of the distribution (*μ*) can be interpreted as the estimated effect size, whereas the width of the distribution (*σ*) indicates the uncertainty about the effect after seeing the data. The Bayes Factor is the ratio of evidence for two opposing hypotheses, defined by the prior and posterior, and usually reflect the alternative‐, and the null‐hypothesis. So, a Bayes Factor of 4.0, indicates that the alternative hypothesis is four times more likely than the null hypothesis given the data at hand, a Bayes Factor of 0.25 (1/4) indicates the opposite (for a more in dept explanation about Bayesian statistics, please see: Wagenmakers et al., 2010) Bayes Factors between 0.3 and 3 are defined as undecisive, not reflecting enough evidence for either hypothesis (Jarosz & Wiley, 2014).

In our Bayesian replication analyses, Bayes Factors of the original study (BForiginal) were computed based on the reported summary statistics (means and standard deviations; Lever & Geurts, 2016) using a flat prior. As aforementioned, BFcurrent is computed using the current data and a flat prior. As such, the results of the original study (BForiginal) could be directly compared to the results of the current study (BFcurrent). Both Bayes Factors indicate whether a group difference is more likely than no group difference or vice versa. Second, “replication Bayes‐Factors” (BFreplication) were computed. The replication BF uses the posterior distribution from the original study as a prior, which together with the current data creates a posterior distribution. In this way the replication BF assesses whether the data in the current study are more likely given the original study (Hreplication) as compared to the effect under H0 (i.e., no difference between groups). In this way, BFreplication indicates whether the original study was replicated by the current study or that the current data are more likely under H0. Third, a “meta‐analytic Bayes Factor” (BFmeta) was computed which pools the data from the two studies together, and uses a flat prior. In this way, the BFmeta reflects the combined effect of the two studies, on the presence or absence of a group difference, which can be directly compared to the separate effects of the two studies. So, in short, BFcurrent and BForiginal reflect the separate effects of the current and the previous study, and BFmeta combines these effects. BFreplication indicates the similarity of the original and current results. For details of the performed analyses, please see the R‐markdown file containing all analyses syntax included as Supporting Information.

## RESULTS

After matching for age, sex, and IQ the sample consisted of 176 adults (88 *n*_autism_, 88 *n*_comparison_). Demographic and symptom characteristics can be found in Table 2. The sample was generally highly educated. In both groups, the majority completed senior secondary education, vocational school, or university (autism: 70%, comparisons: 89%). The autism group had lower levels of education than comparisons. As the groups were matched on IQ, and no correction for education was performed in the original study, no correction was performed to account for this difference. In the autism group, 19 participants (21.5%) scored below the rather stringent ADOS‐2 cut‐off (total score <9). Excluding these participants did not change the pattern of results. Means and standard deviations of the total autism group, and those who scored above the ADOS cut‐off hardly differed (see Table S1).

**TABLE 2:** Demographic and symptom characteristics in our matched sample

Measure	Group			
	Autism (*n* = 88)	Comparison (*n* = 88)	Statistics	
Sex (M/F/O, M %)	54/33/1, 61.4%	54/34/0, 61.4%	*χ* ^2^ = 1.02, *p* = 0.60	
Education^a^	24/29/34	10/38/40	*χ* ^2^ = 7.46, *p* = 0.02	

	Mean (SD); Range	Mean (SD); Range	*t* (*p*)	*d*
Age^b^	55.2 (13.9); 31–85	55.7 (14.4); 30–85	−0.23 (0.82)	−0.04
IQ^c^	115 (15); 85–147	114.4 (14.6); 73–144	0.26 (0.80)	0.04
MMSE^d^	28.9 (1.2); 25–30	29 (1.0); 27–30	−0.68 (0.50)	−0.09
AQ^e^	35.5 (6.6); 15–48	13.4 (6.2); 2–30	22.91 (<0.001)	3.45
ADOS‐2^f^	11.4 (3.8); 2–19			

*Note*: M, men; F, female; O, other.

^a^ Level of education was determined by the Verhage Coding System (Verhage, 1964), between slashes: junior secondary or practical education/senior secondary education or vocational college/university degree.

^b^ Age is provided in decimals.

^c^ IQ was estimated by using two subtests (matrix reasoning and vocabulary) of the Wechsler Intelligence Scale‐IV (WAIS‐IV; Wechsler, 2003).

^d^ Mini Mental State Examination (MMSE; Kok & Verhey, 2002; Folstein et al., 1975) measured global cognitive impairments.

^e^ Autism Quotient (AQ) measured self‐reported autism characteristics.

^f^ Autism Diagnostic Observation Schedule‐2 (ADOS‐2; Lord et al., 2012) was used to verify the autism diagnoses.

### Group differences on our outcome measures

**FIGURE 1:**
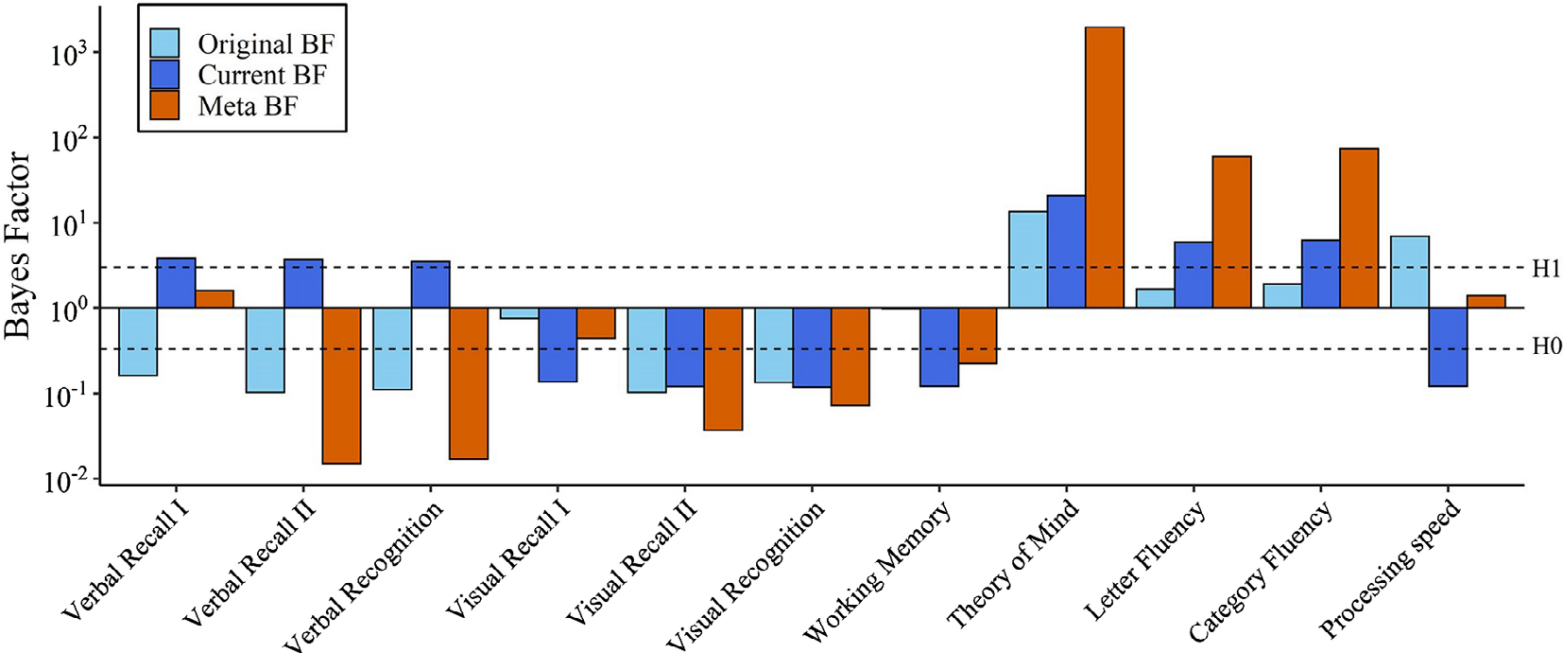
Log‐scaled Bayes factors (BFs) of group differences. Original BFs are Bayesian group differences in the original study using a uniform prior. Current BFs are Bayesian group differences in the current study using a uniform prior. Meta BFs are the pooled group differences of the current, and original study, using a uniform prior. Group differences indicated worse performance of the autism group than comparisons on all variables, except for visual recall I and II

MANOVA analyses indicated poorer performance for the autism group than comparisons on fluency and verbal memory, and equal performance on visual memory. As can be seen in Table 3, *t*‐tests, and their non‐parametric equivalents indicated poorer performance for the autism group on ToM, but not on visual working memory and processing speed. Hence, findings of the original study were replicated on verbal fluency, ToM, visual working memory, delayed visual recall (recall II), and visual recognition. Unexpectedly, comparisons outperformed the autism group on all three outcomes of verbal memory—albeit not when following non‐parametric results. Also, the previously reported group differences of better performance of the autism group in visual immediate recall (recall I) and poorer performance of the autism group in processing speed were not replicated. Finally, as in the original study, more self‐reported cognitive failures were observed in the autism group than in comparisons. BFcurrent confirmed these frequentist results (see Figure 1, dark blue bars).

**TABLE 3:** Group means, standard deviations (SD), and statistics on the cognitive tests and CFQ

	Autism	Comparison	Statistics			
	Mean (*SD*)	Mean (*SD*)	*d*	*t*‐Value	*W*‐value	Replication (BF)
Verbal^a^ Recall I	44 (11.1)	48.1 (8.7)	−0.41	**−2.71** ^**^	**3127** ^*^	**Y** ^b^ (10.3)
Recall II	9.0 (3.3)	10.2 (2.9)	−0.39	**−2.70** ^**^	**3080** ^*^	**Y** ^b^ (3.0)
Recognition	28.3 (2.7)	29.1 (1.2)	−0.38	**−2.68** ^**^	3349	**U** (1.9)
Visual^c^ Recall I	86.4 (12.3)	85.4 (11.8)	0.08	0.55	4120	**U** (0.6)
Recall II	71.8 (2.9)	71.1 (21.9)	0.03	0.22	3937	**U** (0.8)
Recognition	44.7 (2.3)	44.8 (2.4)	−0.04	−0.15	3683	**U** (0.7)
Working memory	0.9 (0.1)	0.9 (0.1)	0.00	0.31	3800	**N** (0.2)
Theory of Mind	26.9 (6.3)	29.6 (4.4)	−0.50	**−3.32** ^***^	**2904** ^**^	**Y** (147.6)
Fluency^d^ Letter	37.1 (11.1)	41.7 (9.9)	−0.44	**−2.88** ^**^	**2897** ^**^	**Y** (36.8)
Category	41.1 (8.5)	44.9 (8.7)	−0.44	**−2.90** ^**^	**2858** ^**^	**Y** (39.5)
Processing speed	422.2 (64.1)	419.7 (66.8)	0.04	0.25	3943	**N** (0.2)
Subjective cognition	46.9 (15.0)	30.1 (9.0)	1.36	**8.96** ^**^	**6357.5** ^**^	**Y** (1.7 × 10^14^)

*Note*: Y, yes; N, No; U, Undecided; ^*^ *= p* < 0.05; ^**^ *= p* < 0.01; ^***^ = *p* < 0.001. Bold values are <.05

^a^ MANOVA overall test: *F*(3,172) = 3.21, *p* = 0.02.

^b^ Please note that even though the current data was better reflected by the original study (*H*_replication_) than *H*_0_, when inspecting the results more carefully, it becomes clear that both hypotheses do not reflect the current data well (see Figure 1).

^c^ MANOVA overall test: *F*(3,171) = 0.20, *p* = 0.89.

^d^ MANOVA overall test: *F*(2,171) = 5.87, *p* = 0.05.

### Bayesian comparisons between the current and the original study

Next to using Bayesian analyses to confirm the current frequentist results, Bayesian analyses were used to compare the results of the current study to the original study. BFreplication indicated that the initial group differences of the previous study were replicated on ToM, and fluency, but also on verbal memory (Table 3). The latter might seem counterintuitive as the original study did not find a group difference on verbal memory. However, BFreplication merely quantifies the relative evidence for *H*_replication_ compared to *H*_0_. So, BFreplication indicates that the current data were better explained by the original study compared to *H*_0_, but not necessarily that the current findings are similar to the original study (the so called: “replication paradox,” Ly et al., 2019). For working memory and processing speed, *H*_0_ (no difference) gives a better account of the data than the original study_._ This is also reflected by the differences between the current and original study on these domains, most notable on processing speed (see dark‐ and light blue bars in Figure 1). For verbal recognition and visual memory, BFreplication was undecisive, indicating neither evidence for successful nor failed replication.

In line with the BFreplication, BFmeta, combining the effects of both studies, indicated a substantial effect in favor of a group difference for verbal fluency, and ToM, see Figure 1 (orange bars). Substantial evidence for *H*_0_ (no differences) was observed on verbal‐ and visual delayed recall (recall II), verbal‐ and visual‐recognition and working memory. BFmeta were undecisive for direct verbal‐ and visual recall (recall I), and processing speed. All Bayes Factors are provided in Table S2.

### Age‐related differences

**FIGURE 2:**
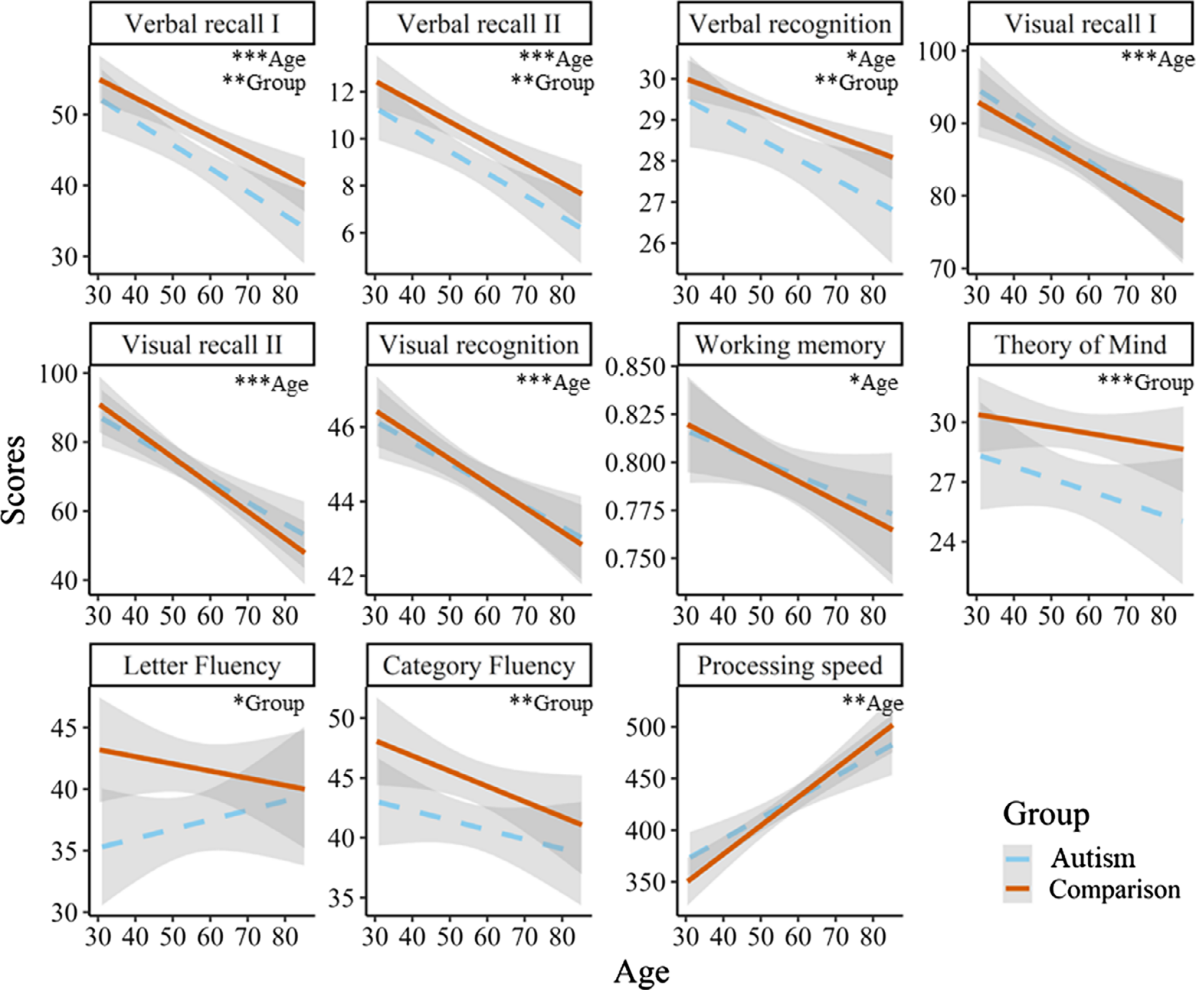
A visual display of the regression analyses containing group, age, and their interaction as predictors. Higher scores indicate better performance on all variables, except for processing speed. Asterisks indicate significant effects of group and age (* = *p* < 0.05; ** = *p* < 0.01; *** = *p* < 0.001). Age x group effects were all nonsignificant

Regression analyses—including (centered) age, group, and their interaction—confirmed group differences on fluency, ToM and verbal memory, see Table 4. Also, a significant relation between older age and poorer performance was observed on verbal memory, visual (working) memory, and processing speed. Unexpectedly, no age effects were observed on ToM, and fluency. The predominantly parallel aging pattern between both groups was replicated, as no significant interactions between age and group on any of the outcomes were observed, see also Figure 2. Unexpectedly, the protective effect of autism on visual learning and visual working memory were not replicated. Including age^2^ and age^2^ x group as predictors only improved the models marginally (*R* ^*2*^ max difference = 0.02) or not at all. Results can be found in Table S3.

**TABLE 4:** Regression coefficients for cognitive test outcomes with age, group and their interaction containing as predictors

	Predictors			Fit index
	Age	Group	Age x Group	*R* ^2^
Verbal Recall I				
*β*	**−0.27**	**−4.25**	−0.06	0.22
*t*	**−4.01** ^***^	**−3.10** ^**^	−0.62
Recall II				
*β*	**−0.09**	**−1.30**	−0.01	0.21
*t*	**−4.17** ^***^	**−3.05** ^**^	−0.20
Recognition				
*β*	**−0.03**	**−0.87**	−0.01	0.12
*t*	**−2.33** ^*^	**−2.86** ^**^	−0.66
Visual Recall I				
*β*	**−0.30**	0.85	−0.03	0.14
*t*	**−3.55** ^***^	0.50	−0.29
Recall II				
*β*	**−0.78**	0.41	0.16	0.22
*t*	**−5.56** ^***^	0.14	0.78
Recognition				
*β*	**−0.07**	−0.07	0.01	0.14
*t*	**−3.97** ^***^	−0.21	0.36
Working Memory				
*β*	**<0.01**	<0.01	<0.01	0.04
*t*	−2.21*	0.26	0.34
Theory of Mind				
*β*	−0.03	**−2.77**	−0.03	0.07
*t*	−0.78	**−3.36** ^***^	−0.49
Fluency Letter				
*β*	−0.06	**−4.53**	0.13	0.05
*t*	−0.74	**−2.84** ^*^	1.19
Category				
*β*	−0.13	**−3.83**	0.05	0.08
*t*	−2.01	**−2.96** ^**^	0.52
Processing speed				
*β*	**2.76**	3.07	−0.74	0.28
*t*	**6.60** ^**^	0.36	−1.23

*Note*: ^*^ *= p* < 0.05; ^**^ *= p* < 0.01; ^***^ = *p* < 0.001. Bold values are <.05. Groups were autism versus comparison. Fit was compared to the fit indices in the models using age^2^ (Table S1) and in the 50+ sample (Table S3). *R* ^2^ was used as the indicator for model fit (higher = better fit).

Abbreviations: AIC, Akaike Information Criterion; BIC, Bayesian Information Criterion.

### 50+ sample

To assess whether the aforementioned results might show a different pattern if analyzed separately in the older adult sample, the data were reanalyzed in adults of 50 years and older (*n*_autism_ = 56, *n*_comparison_ = 57). In the 50+ sample, group differences were generally similar, but of a smaller magnitude, as compared to the differences observed in the full sample, with two exceptions. First, on verbal fluency, BFcurrent did not indicate a group difference in the 50+ sample (see Figure S1). As BFmeta still indicated substantial evidence in favor of a group difference, this seems to be due to a lack of power in the 50+ sample. Second, on immediate recall visual memory (recall I), BFmeta indicated a group difference, with autistic individuals outperforming comparisons. This group difference seems to be driven fully by the results of the previous study, considering that BFcurrent indicated substantial evidence for *H*_0_ (no group difference). For the regression analyses, no major differences with the full sample were observed. Full statistical details on these group‐ and regression analyses can be found in Tables S4 and S5.

## DISCUSSION

In the current study, no evidence for altered age‐related cognitive effects in adults with autism was observed. Hence, evidence for a parallel aging pattern was replicated in which age‐related cognitive effects are similar in autistic and non‐autistic adults. A pattern in which initial differences diminish at older age (“protective aging”) was not replicated. Furthermore, poorer performance of autistic adults was observed on two domains, namely verbal fluency (generativity) and ToM, which were both not associated with age. Hence even on domains where autistic adults showed difficulties, results do not indicate that autistic adults are at risk for cognitive difficulties at older age specifically.

These age‐related effects were in line with previously observed parallel aging patterns on verbal memory, fluency, and processing speed (Ambery et al., 2006; Geurts & Vissers, 2012; Lever & Geurts, 2016; Tse et al., 2019), and studies of middle aged to older autistic adults that did not observe major group differences on these domains (Braden et al., 2017; Spek et al., 2010). Furthermore, Bayesian results that combined the data from the current and original study showed substantial evidence for equal performance between groups on delayed verbal, and visual memory, verbal and visual recognition, and visual working memory. Although, evidence for verbal memory was contradictive between the current and original study, as the current study indicated evidence for diminished performance in the autism group, but the original study did not indicate a group difference. The data of the two studies combined convincingly indicate that it is about 50 times more likely that no group differences exist on verbal memory. Given that memory domains are generally sensitive to cognitive aging (Salthouse, 2004), data from the combined studies further supported the hypothesis that cognitive aging is not specifically different in autism. These results are in contrast to studies suggesting that autistic adults might be at risk for accelerated cognitive decline (Koolschijn et al., 2017; Powell et al., 2017; Torres et al., 2020; Walsh et al., 2019). However, most of these studies used indirect measures of cognition (i.e., brain or motion), and had rather small sample sizes, limiting the comparability to the current study. The current results are also in contrast to epidemiological studies, which might indicate a risk for accelerated (cognitive) aging in autism (Bishop‐Fitzpatrick & Rubenstein, 2019; Croen et al., 2015; Hand et al., 2020; Rydzewska et al., 2018). Arguably, the current study might not have captured those most “at risk” for accelerated cognitive decline, because our sample was limited to those who could actively participate in a study, and those with a neurological disorder were excluded (*n*_autism_ = 28, *n*_comparisons_ = 9).

Protective age‐related effects on visual working memory and ToM were not replicated (Lever et al., 2015; Lever & Geurts, 2016). On visual working memory and ToM, similar performance of autistic and non‐autistic 50+ adults were observed, also when taking the previous study into account. So, in light of the current findings, the previous findings on compensatory abilities of autistic adults at older age on these domains should be reconsidered. In visual working memory, a study by Tse et al. (2019) also reported no differential age‐effect. They argued that the work by Lever et al. (2015) showed enhanced protective aging effects anticipating on the strengths of autistic adults. As the current study used the same task, but did not observe a differential age‐effect, the current findings nuance such an explanation, and are in line with studies that also observed non‐differential age‐effects (Abbott et al., 2018; Braden et al., 2017). In ToM, evidence on protective age‐related effects has been observed using a composite score of several ToM measures, but only when assessing age categorically (i.e., younger vs. older than 50 years; Zivrali Yarar et al., 2020). As no general effect of age was observed in the current study, and age effects are still debated in ToM also in non‐autistic adults (Bottiroli, 2016; Charlton, Barrick, Markus, & Morris, 2009; Happé & Winner, 1998; Moran, 2013), longitudinal research seems a vital next step to further investigate the aging patterns on this domain.

A protective aging effect of autism was also not replicated on direct visual memory. Data from the two studies combined, indicated modest evidence for better performance of autistic 50+ adults, than non‐autistic 50+ adults on direct visual memory, but data from the current study only were not in line with this observation. Therefore, the data from the previous study seem predominant in this effect, and in contrast with data from the current sample. As the current 50+ sample is approximately 5 years older than the previous sample, the current study had more data available on the final stage of adulthood. Therefore, one would expect that a true protective effect of autism would even become more evident using only the current data. Possibly, the limited number of older participants in the previous sample represented those who perform rather well on visual memory, biasing the results to be more positive. Another possibility might be that these differences reflect a protective effect until a certain point, hinting at delayed decline of visual memory inautism. Given the contrasting nature of these findings and contrasting results in other studies (Geurts & Vissers, 2012; Ring et al., 2016; Tse et al., 2019), combined with a lack of longitudinal evidence, conclusions on visual memory remain inconclusive.

The results of the current study should be viewed in light of some strengths and limitations. The replicative nature of our study by carefully assessing previous findings before going into new directions, is valuable in a field where large heterogeneity exists between studies. Also, the inclusion of Bayesian analyses showed that not all results were confirmed when taking prior knowledge into account. Current developments within the Bayesian field might guide us to further optimize the use of prior knowledge in future work. More specifically, the current meta‐analytic Bayes Factors could be used as a prior for future studies using similar tasks. Limitations are threefold. First, the representativeness of the current sample is limited to (a) those who could actively participate in research (b) those without intellectual disabilities, and (c) primarily those who received their diagnosis in (late) adulthood. A portion of the autistic participants did not score above the ADOS‐2 cut‐off. This is, given the instrumental issues of this measure (Bastiaansen et al., 2011), not unexpected. As all participants had a clinical diagnosis, were often recruited via clinical care, reported various impairments, and generally scored high on autism‐related measures, we regard this sample, although equally valuable, dissimilar to studies describing broad autism phenotype (BAP) samples, (Stewart et al., 2018, 2020; Wallace et al., 2016). Also, while the sample includes more older participants than most studies on cognitive aging in autism, it was difficult to recruit the oldest age group (70+) with autism, particularly women. Therefore, the current study was limited in investigating sex specific age‐related cognitive effects. Whether age‐related cognitive effects are indeed largely parallel in autistic men and women remains an important question that needs to be addressed in future research.

Second, our study aimed at capturing those cognitive domains most sensitive to aging (i.e., various forms of memory, verbal ability, and processing speed) and/or autism (ToM) but might have underrepresented domains of executive functioning (EF), as only included verbal fluency and working memory were included. More work on EF in older autistic adults will be a valuable addition to the current results as it might provide a more exhaustive image of cognitive functions imperative to daily living.

Third, the current results are limited by cross‐sectional data, and the findings could be biased by cohort effects. Given that diagnostic criteria of autism changed over time (e.g., DSM‐III to DSM‐5), and differences in diagnostic age within the sample, these findings of mainly parallel age‐related cognitive effects need longitudinal replication.

In sum, the most likely cognitive aging scenario of autistic adults is one of parallel aging. This implies that difficulties present in young adulthood are still present at older age but might also imply that strengths do not diminish at a faster pace. Contrary to beliefs of faster cognitive aging, these results might come as a relief for those autistic adults worrying about their cognitive abilities at older age.

## CONFLICT OF INTEREST

The authors declare no conflict of interest. The study was approved by the ethical review board of the Department of Psychology of the University of Amsterdam (2018‐BC‐9285).

## AUTHOR CONTRIBUTIONS

All authors contributed to designing the study, analyses and pre‐registration. Carolien Torenvliet, Tulsi A. Radhoe and Wikke J. Van der Putten were responsible for data collection. Carolien Torenvliet performed the analyses, and wrote the first draft. Carolien Torenvliet, Annabeth P. Groenman, and Hilde M. Geurts were involved in feedback on subsequent versions. All authors provided feedback on the final version and approved the manuscript.

## Supporting information


**Appendix S1**: Supporting Information

## Footnotes

^1^
Variability in terminology to describe individuals with autism/autistic individuals is deliberately used, to reflect the wide variability of preferences within the autism community (Kenny et al., 2016).

^2^
None of our participants scored below 25 on the MMSE.

^3^
Note that the original study reported *F*‐values, corresponding to a one‐way ANOVA. However, with two groups the ANOVA and *t*‐test yield exactly the same results (*t* = √*F*). We chose to report t‐values as they directly correspond to the Bayesian replication method.
