# Supplementary material for: Parallel age‐related cognitive effects in autism: A cross‐sectional replication study
Source: Autism Res. 2021 Dec 4;15(3):507–18. doi: 10.1002/aur.2650 (PMC9300037; doi:10.1002/aur.2650)
Supplement: Supplementary file 1 — Appendix S1: Supporting Information [file AUR-15-507-s001.docx]

**Supplementary materials**

**Description of differences between the original and replication (current) study**

Differences were 1) age range is slightly shifted (replication: 30-89 years, original: 20-79 years), 2) the Intelligence Quotient (IQ) was estimated with an updated version of the Wechsler Adult Intelligence Scale (WAIS; replication: WAIS-IV [Wechsler, 2008] original: WAIS-III [Wechsler, 1997a]), 3) autism diagnoses were validated using an updated version of the Autism Diagnostic Observation Schedule (ADOS; replication: ADOS-2 [Lord et al., 2012], original: ADOS [Lord et al., 2000)]. 4) the absence of Attention Deficit Hyperactivity disorder (AD[H]D) in the comparison group was further validated using the ADHD Rating Scale (ADHD-SR, [Kooij, Buitelaar, Furer, Rijnders, & Hodiamoent, 2005]), 5) the exclusion criterium for IQ was slightly more lenient (replication: IQ>69, original: IQ>79), 6) for the comparison group an adjustment to the procedure was made to make data collection more efficient. If preferred by the participant, the interview session was administered directly after the cognitive session.

**ADOS+ Sample**

**Table S1**

*Group means, standard deviations (SD) for the main outcome variables in the total autism group and those that scored above the ADOS-2 cut-off.*

|  | | | Autism (N=88) | ADOS+ (N=69) |
| --- | --- | --- | --- | --- |
|  | | | Mean (SD) | Mean (SD) |
| Age | | | 55.2 (13.9) | 55.3 (14.4) |
| MMSE | | | 28.9 (1.2) | 29.0 (1.1) |
| IQ | | | 115.0 (15) | 116.4 (14.6) |
| Verbal^a^ Recall I | | | 44.0 (11.1) | 43.8 (11.6) |
|  | Recall II | | 9.0 (3.3) | 9.0 (3.3) |
|  | Recognition | | 28.3 (2.7) | 28.0 (2.9) |
| Visual^b^ Recall I | | | 86.4 (12.3) | 85.7 (12.9) |
|  | Recall II | | 71.8 (20.9) | 71.0 (21.8) |
|  | Recognition | | 44.7 (2.3) | 44.7 (2.4) |
| Working memory | | | .9 (.1) | .9 (.1) |
| Theory of Mind | | | 26.9 (6.3) | 26.6 (6.5) |
| Fluency^c^ Letter | | | 37.1 (11.1) | 36.9 (11.0) |
|  | | Category | 41.1 (8.5) | 41.0 (8.9) |
| Processing speed | | | 422.2 (64.1) | 428.9 (67.4) |
| Subjective cognition | | | 46.9 (15.0) | 48.0 (14.9) |

**Bayes Factors**

**Table S2.**

Bayes Factors for the original, current, meta-analytic, and replication results.

|  | | BF original | BF current | BF meta | BF replication |
| --- | --- | --- | --- | --- | --- |
| Verbal | Recall I | **0.16 (6.25)** | **3.80** | 1.60 | **10.36** |
|  | Recall II | **0.10 (10.00)** | **3.71** | **0.02 (50.00)** | **3.02** |
|  | Recognition | **0.11 (9.09)** | **3.53** | **0.02 (50.00)** | 1.90 |
| Visual | Recall I | 0.76 (1.32) | **0.14 (7.14)** | 0.44 (2.27) | 0.58 (1.72) |
|  | Recall II | **0.10 (10.00)** | **0.12 (8.33)** | **0.04 (25.00)** | 0.75 (1.33) |
|  | Recognition | **0.13 (7.69)** | **0.12 (8.33)** | **0.07 (14.29)** | 0.71 (1.41) |
| Working memory | | 0.98 (1.02) | **0.12 (8.33)** | **0.22 (4.55)** | **0.23 (4.35)** |
| Theory of Mind | | **13.48** | **20.66** | **1947.08** | **147.72** |
| Fluency | Letter | 1.67 | **5.91** | **59.90** | **36.85** |
|  | Category | 1.92 | **6.23** | **73.84** | **39.43** |
| Processing speed | | 6.93 | **0.12 (8.33)** | 1.40 | **0.19 (5.26)** |

*Note.* BF, Bayes Factor; meta, meta-analytic. For Bayes Factors < 1 evidence for H_0_ is provided between brackets (BF^-1^). BFs of significance (>3 or <.3) are in **bold**.

**Age-squared regressions**

**Table S3**

*Regression Coefficients for Cognitive Test Outcomes with Age^2^, Group and their Interaction Containing as Predictors.*

|  |  |  | Statistics | | | Fit index | |
| --- | --- | --- | --- | --- | --- | --- | --- |
|  |  |  | Age^2^ | Group | Age^2^ x Group | *R*^2^ | Better fit?^a^ |
| Verbal Recall I | | *β* | **<.01** | **-4.31** | <.01 | .22 | Yes |
|  |  | *t* | **-4.03^***^** | **-3.15^**^** | -0.65 |  |  |
|  | Recall II | *β* | **<.01** | **-1.31** | <.01 | .20 | No |
|  |  | *t* | **-4.00^***^** | **-3.08^**^** | -0.30 |  |  |
|  | Recognition | *β* | **<.01** | **-0.87** | <.01 | .13 | Yes |
|  |  | *t* | **-2.31^*^** | **-2.90^**^** | -0.79 |  |  |
| Visual Recall I | | *β* | **<.01** | 0.79 | <.01 | .15 | Yes |
|  |  | *t* | **-3.58^***^** | 0.47 | -0.43 |  |  |
|  | Recall II | *β* | **-0.01** | 0.33 | <.01 | .24 | Yes |
|  |  | *t* | **-5.80^***^** | 0.11 | 0.85 |  |  |
|  | Recognition | *β* | **<.01** | -0.08 | <.01 | .14 | Yes |
|  |  | *t* | **-3.89^***^** | -0.24 | 0.20 |  |  |
| Working memory | | *β* | **<.01** | <.01 | <.01 | .05 | Yes |
|  |  | *t* | **-2.33^*^** | 0.25 | 0.44 |  |  |
| Theory of Mind | | *β* | <.01 | **-2.79** | <.01 | .08 | Yes |
|  |  | *t* | -0.68 | **-3.39^***^** | -0.70 |  |  |
| Fluency Letter | | *β* | <.01 | **-4.52** | <.01 | .05 | No |
|  |  | *t* | -0.70 | **-2.83^**^** | 1.12 |  |  |
|  | Category | *β* | **<.01** | **-3.84** | <.01 | .08 | Yes |
|  |  | *t* | **-2.19^*^** | **-2.98^**^** | 0.63 |  |  |
| Processing speed | | *β* | **0.03** | 3.17 | <0.01 | .28 | No |
|  |  | *t* | **6.65^***^** | 0.37 | -1.45 |  |  |

*Note*. *^*^=p*<.05; *^**^=p*<.01; ^***^=*p*<.001^a^ Fit was compared to the fit indices of linear age. *R^2^* was used as the indicator for model fit (higher = better fit).

**Group differences in our 50+ sample**

**Table S4**

*Group means, standard deviations (SD), and Statistics on the Cognitive Tests and CFQ in our 50+ Sample.*

|  | | | Autism | Comparison | Statistics | |  |  |
| --- | --- | --- | --- | --- | --- | --- | --- | --- |
|  | | | Mean (SD) | Mean (SD) | *d* | *t* (*p*) | *W* (*p*) | Replication (BF) |
| Verbal^a^ Recall I | | | 40.9 (11.2) | 46 (8.1) | -.52 | **-2.75^**^** | **1174.5^*^** | **U** (1.5) |
|  | Recall II | | 8.0 (3.1) | 9.5 (2.7) | -.52 | **-2.69^**^** | **1200.0^*^** | **N** (0.2) |
|  | Recognition | | 27.9 (3.1) | 28.8 (1.3) | -.38 | -1.94 | 1552.0 | **N** (0.2) |
| Visual^b^ Recall I | | | 84.7 (13.6) | 82.6 (12.5) | .16 | .83 | 1788.0 | **N** (0.3) |
|  | Recall II | | 67 (21.4) | 65.2 (23.2) | .08 | .44 | 1664.0 | **U** (0.7) |
|  | Recognition | | 44.4 (2.3) | 44.3 (2.5) | .04 | .24 | 1553.5 | **U** (0.5) |
| Working memory | | | 0.9 (0.1) | 0.9 (0.1) | .00 | .27 | 1546.5 | **N** (0.1) |
| Theory of Mind | | | 26.3 (6.2) | 29.3 (4.6) | -.55 | **-3.0^**^** | **1140.0^**^** | **Y** (21.6) |
| Fluency^c^ Letter | | | 38.2 (11.2) | 41.5 (8.9) | -.33 | -1.7 | 1286.0 | **U** (2.7) |
|  | | Category | 40.9 (8.8) | 44.4 (9.1) | -.39 | **-2.08^*^** | 1211.5 | **Y** (5.9) |
| Processing speed | | | 444.4 (60.2) | 442.5 (66.6) | .03 | .16 | 1604.5 | **U** (0.6) |
| Subjective cognition | | | 46.5 (15.5) | 30.4 (9.0) | 1.27 | **6.77^**^** | **2580.5^**^** | **U** (1.5) |

*Note.* BF, Bayes Factor; Y, yes; N, No; U, Undecided; ^*^=p<.05; ^**^=p<.01; ^***^=p<.001.

^a^ MANOVA overall test: *F*(3,109)=2.71, *p*=.05;

^b^ MANOVA overall test: *F*(3,108)=.26, *p=*.86;

^c^ MANOVA overall test: F(2,108)=2.42, p=.09.

**Figure S1**

*Log-Scaled Bayes Factors (BFs) of Group Differences in our 50+ Sample.*

*
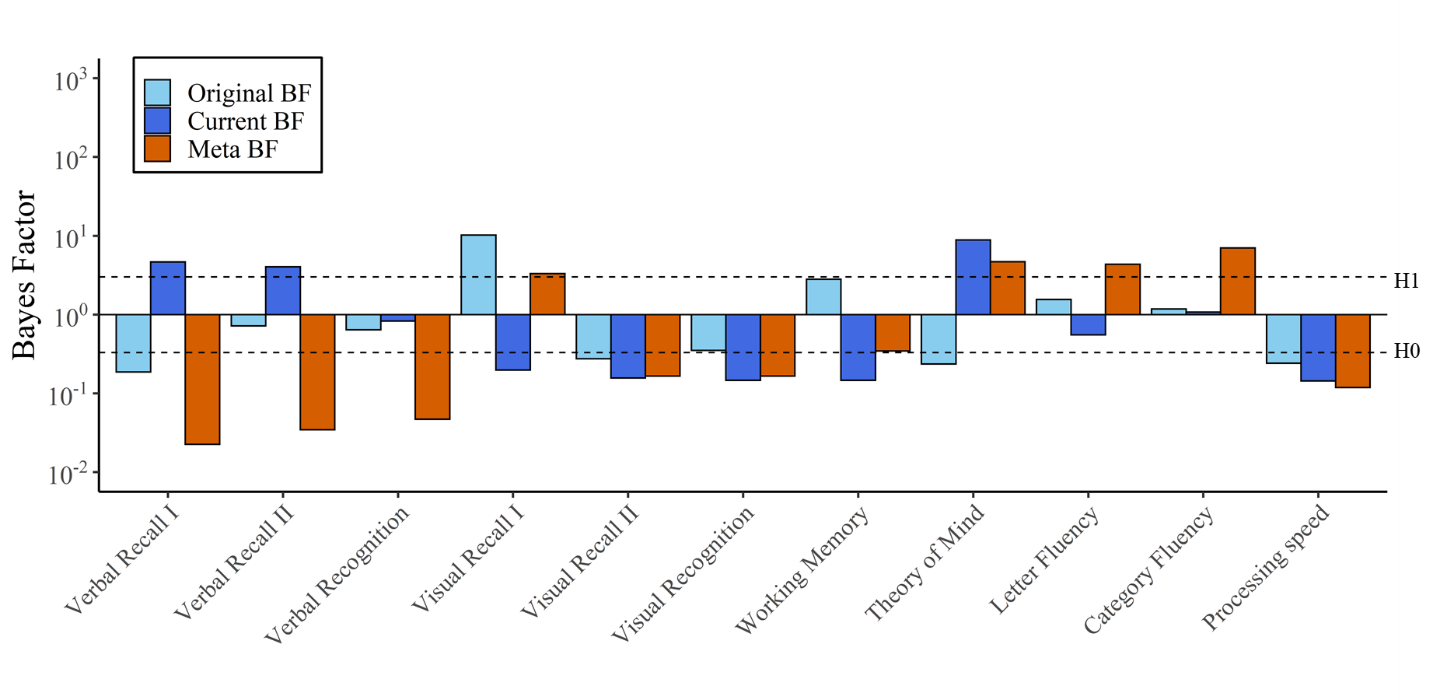
*

**Table S5**

*Regression Coefficients for Cognitive Test Outcomes with Age, Group and their Interaction Containing as Predictors in our 50+ Sample.*

|  |  |  | Statistics | | | Fit index | |
| --- | --- | --- | --- | --- | --- | --- | --- |
|  |  |  | Age | Group | Age x Group | *R^2^* | Better fit?^a^ |
| Verbal Recall | | *β* | **-.38** | **-5.34** | <.01 | .16 | No |
|  |  | *t* | **-2.43^*^** | **-2.08^*^** | -.01 |  |  |
|  | Recall II | *β* | -.07 | -1.47 | -.01 | .10 | No |
|  |  | *t* | -1.49 | -1.86 | -.10 |  |  |
|  | Recognition | *β* | -.03 | -.42 | -.06 | .09 | No |
|  |  | *t* | -.79 | -.65 | -1.07 |  |  |
| Visual Recall | | *β* | -.36 | 4.57 | -.34 | .13 | No |
|  |  | *t* | -1.71 | 1.34 | -1.19 |  |  |
|  | Recall II | *β* | **-1.37** | -2.31 | .37 | .19 | No |
|  |  | *t* | **-3.98^***^** | -.41 | .79 |  |  |
|  | Recognition | *β* | **-.08** | .35 | -.04 | .11 | No |
|  |  | *t* | **-1.97^*^** | .56 | -.68 |  |  |
| Working memory | | *β* | **<.01** | -.01 | <.01 | .06 | No |
|  |  | *t* | **-2.37^*^** | -.57 | .88 |  |  |
| Theory of Mind | | *β* | <.01 | -2.02 | -.13 | .09 | Yes |
|  |  | *t* | .04 | -1.35 | -1.01 |  |  |
| Fluency Letter | | *β* | -.05 | -3.68 | .05 | .03 | No |
|  |  | *t* | -.32 | -1.31 | .20 |  |  |
|  | Category | *β* | **-.40** | **-5.53** | .21 | .12 | Yes |
|  |  | *t* | **-2.75^**^** | **-2.30^*^** | 1.02 |  |  |
| Processing speed | | *β* | **3.94** | 26.98 | -2.72 | .14 | No |
|  |  | *t* | **3.88^***^** | 1.63 | -1.95 |  |  |

*Note*. *^*^=p*<.05; *^**^=p*<.01; ^***^=*p*<.001. ^a^ Fit was compared to the fit indices in the full sample with the same predictors. *R^2^* was used as the indicator for model fit (higher = better fit).
